# Country-Specific Approaches to Preventing Infections in Cataract Surgery

**DOI:** 10.3390/antibiotics14121192

**Published:** 2025-11-23

**Authors:** Mario Damiano Toro, Alina Popa-Cherecheanu, Nora Majtanova, Štěpán Rusňák, Nikoloz Labauri, Vladimir Pfiefer, Nikolai Dakov, Gábor Németh, Vahe Nanyan, Izabela Korona-Głowniak, Robert Rejdak

**Affiliations:** 1 Department of General and Pediatric Ophthalmology, Medical University of Lublin, 20-079 Lublin, Poland; 2 Eye Clinic, Public Health Department, Federico II University, 80138 Naples, Italy; 3 Department of Ophtalmology, Carol Davila University of Medicine and Pharmacy, 050474 Bucharest, Romania; 4 Department of Ophthalmology, University Emergency Hospital, 050098 Bucharest, Romania; 5 Department of Ophthalmology, Slovak Medical University and University Hospital in Bratislava, 85107 Bratislava, Slovakia; 6 Department of Ophthalmology, Faculty of Medicine in Pilsen, Charles University in Prague and University Hospital in Pilsen, 32300 Prague, Czech Republic; 7 Center of Eye Microsurgery Ofta, 32300 Pilsen, Czech Republic; 8 DAVINCI Eye Clinic, 0194 Tbilisi, Georgia; 9 Eye Hospital, University Clinical Center Ljubljana, 1000 Ljubljana, Slovenia; 10 Department of Ophthalmology, Medical University of Sofia, 1431 Sofia, Bulgaria; 11 Faculty of Health Sciences, Institute of Clinical Methodology, University of Miskolc, 3515 Miskolc, Hungary; 12 Nanyan Eye Microsurgery Center, Yerevan 0054, Armenia; 13 Department of Pharmaceutical Microbiology, Medical University of Lublin, 20-093 Lublin, Poland

**Keywords:** antibiotics, antibiotic resistance, cataract, prophylaxis, surgery

## Abstract

Background/Objectives: Antimicrobial resistance (AMR) is a major global health threat. In patients undergoing cataract surgery, AMR complicates infection control, particularly efforts to reduce the risk of endophthalmitis—a rare but severe postoperative complication. This article reviews country-specific strategies for endophthalmitis prevention, focusing on antimicrobial use. Methods: Ophthalmology experts from 10 countries contributed national perspectives on infection prevention. Official guidelines served as the primary basis for analysis; when unavailable, expert opinion and routine clinical practice were considered. Results: Routine preoperative antibiotic use is uncommon in 6 out of 10 countries. Instead, artificial tears and bacteriostatic or bactericidal treatments are frequently employed. One country allows optional antibiotic use, while 3 include it in routine preoperative care. For intraoperative management, antisepsis with 5–10% povidone-iodine is standard practice in 9 countries. Intracameral cefuroxime (typically 1 mg/0.1 mL) is widely used in 7 countries and considered essential in most countries. Postoperatively, broad-spectrum topical antibiotics, primarily fluoroquinolones, are typically prescribed, often as fixed-dose combinations with corticosteroids (8 countries), although duration and regimens vary. Conclusions: Despite national differences, povidone-iodine, intracameral cefuroxime, and topical fluoroquinolones are widely used. Preoperative antibiotic use varies, while postoperative regimens are more consistent. These practices reflect local AMR patterns, regulations, and available healthcare resources. Although broad-spectrum agents are generally preferred, they raise concerns about resistance. Tailoring prophylactic strategies to local microbiological profiles and limiting the duration of antibiotic therapy are key to balancing efficacy and stewardship. An individualized, evidence-based approach is essential to reduce endophthalmitis risk and address AMR challenges.

## 1. Introduction

Antimicrobial resistance (AMR) is recognized by the World Health Organization (WHO) as one of the top 10 global public health threats. In 2019, it was responsible for 1.27 million deaths globally and was associated with an additional 4.95 million deaths [1]. Although AMR-related mortality slightly declined in 2021, projections suggest that the global number of AMR-attributable deaths will increase by 13.4% by 2030 and by 69.6% by 2050 [2].

AMR is a growing concern across various clinical settings, including in patients undergoing cataract surgery. The emergence of resistant bacterial strains has significantly elevated the risk of postoperative ocular infections. Cataract surgery is one of the most common, effective, and successful medical interventions [3], although its rate varies widely between countries, ranging from 36 to 12,800 procedures per million population [4]. In 2021, the global prevalence of cataract was estimated at 101 million cases, with the overall burden expected to rise in the coming years [5].

Cataract surgery can be complicated by endophthalmitis, with the risk increasing in older patients, males, and individuals with comorbidities such as diabetes, hypertension, malignancies, and a history of stroke. Endophthalmitis is typically caused by Gram-positive bacteria, often originating from the patient’s eyelid flora [6,7]. Although its incidence following cataract surgery is relatively low—ranging from 0.04% to 0.3%—it remains one of the most serious postoperative complications due to its potentially sight-threatening consequences [8,9]. Given the severity of this condition, infection prevention in cataract surgery is critically important. The growing challenge of AMR further emphasizes the need for robust antibiotic stewardship and individualized prophylactic strategies. As resistance patterns vary geographically, effective prevention should be guided by local microbiological data.

The aim of this article was to summarize country-specific strategies for preventing endophthalmitis following cataract surgery, with a focus on antimicrobial measures.

## 2. Results

**Figure 1:**
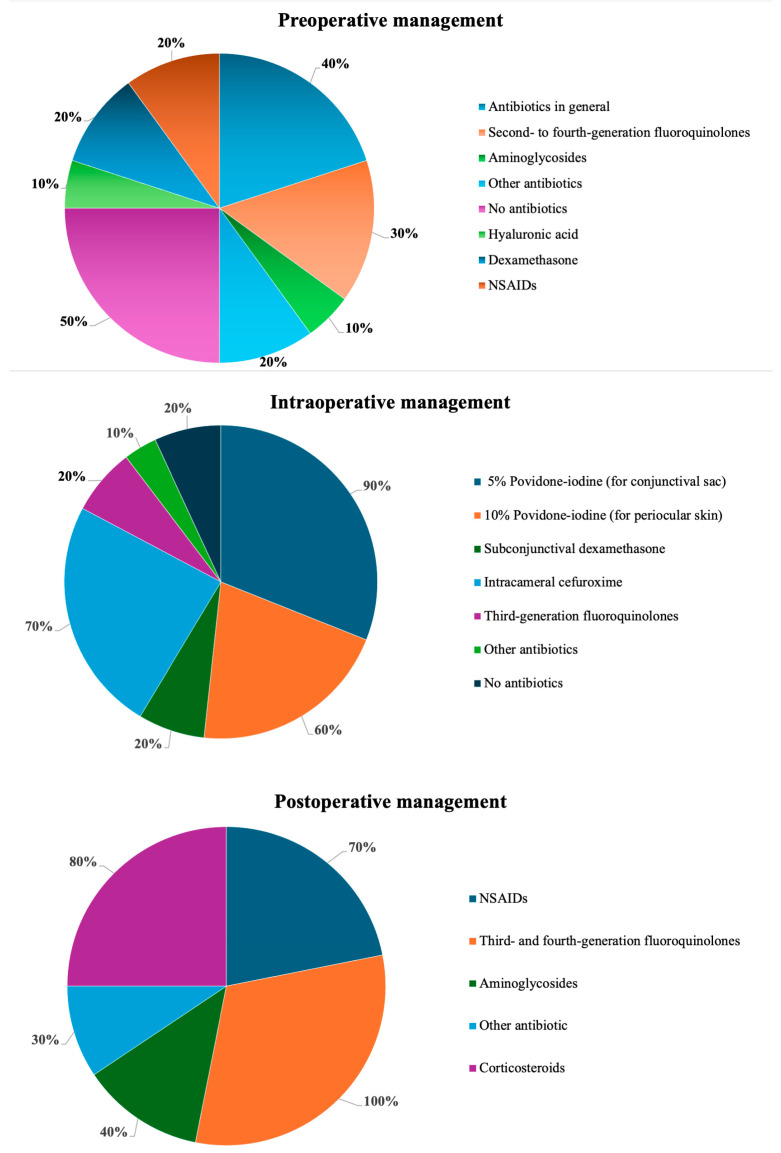
Summary of approaches at various stages of infection prophylaxis in cataract surgery.

A brief summary is provided in Figure 1, while detailed descriptions are presented in the main text and in Supplementary Table S1.

### 2.1. Preoperative Management

Although experts generally recognize the importance of infection prevention in cataract surgery, approaches—particularly regarding preoperative antibiotic use—remain highly variable. In more than half of the countries included in this study (Armenia, Bulgaria, Georgia, Hungary, Italy, and Romania), antibiotics are not administered before surgery. Prophylactic antibiotic use in the days preceding the procedure is generally discouraged due to the risk of promoting bacterial resistance. Instead, preoperative management typically involves the use of artificial tears to address dry eye and bacteriostatic or bactericidal treatments in cases of blepharitis, aimed at reducing the risk of surgical wound contamination [10]. The term “bacteriostatic or bactericidal treatments” refers here to topical antimicrobials or antiseptics used preoperatively to lower bacterial load in patients with blepharitis or ocular surface disease. Common options include short courses of topical azithromycin or fusidic acid and lid hygiene with antiseptic cleansers. These measures aim to reduce eyelid margin colonization and the risk of surgical wound contamination.

In Georgia, patients with pseudoexfoliation syndrome additionally receive nonsteroidal anti-inflammatory eye drops, typically for 10 days before surgery, to maintain pupil dilation and reduce postoperative inflammation.

Polish guidelines state that current scientific evidence does not sufficiently support the routine preoperative use of antibiotics [11]. However, they acknowledge that some authors recommend initiating treatment with selected fluoroquinolones 2 days prior to surgery [11]. Antibiotics are administered preoperatively in Czechia, Slovakia, and Slovenia. In Slovakia, the effectiveness of topical antibiotics (second- to fourth-generation fluoroquinolones, aminoglycosides, or their combinations) given 1 to 3 days before surgery is considered unproven when povidone-iodine antisepsis and 1 mg of intracameral cefuroxime are used at the end of the procedure. Consequently, a combination of levofloxacin and dexamethasone is limited to the day before surgery. In Slovenia, the protocol includes a combination of an antibiotic, dexamethasone, and a nonsteroidal agent administered over 3 days preoperatively. The most liberal approach to preoperative use of topical antibiotics is observed in Czechia, where second- to fourth-generation fluoroquinolones (ofloxacin, levofloxacin, moxifloxacin), aminoglycosides (neomycin, gentamycin, tobramycin), or other combinations of antibiotics are frequently used 1 to 3 days before surgery, despite limited supporting evidence [12].

Despite these variations, there is a broad consensus on the importance of tailoring prophylactic antibiotic regimens to individual patient profiles and local microbial resistance patterns. However, the role and timing of topical antibiotics continue to vary depending on national guidelines and practitioner preferences.

### 2.2. Intraoperative Management

In the countries included in this review, antisepsis is the primary focus at the initiation of cataract surgery, with povidone-iodine serving as a key agent for ocular surface disinfection. A 5% solution is usually instilled into the conjunctival sac, while concentrations ranging from 5% to 10% are applied to the eyelids and surrounding skin and typically left in place for approximately 3 min. In Slovakia, slightly lower concentrations are used: 3–5% for the conjunctival sac and 5–10% for the skin. In contrast, Slovenia limits the maximum povidone-iodine concentration to 5% due to concerns about potential ocular surface toxicity.

The next key pharmacological step in intraoperative management is the intracameral administration of cefuroxime. In most of the countries analyzed, 0.1 mL of solution containing 1 mg of cefuroxime is typically used. Georgia represents an exception, where no intraoperative antibiotics are administered. In the majority of countries, intracameral cefuroxime is regarded as a standard component of endophthalmitis prophylaxis. For example, in the Czech Republic, majority of ophthalmic surgeons use intracameral cefuroxime, while only few ophthalmic surgeons employ alternative intracameral antibiotics or rely exclusively on postoperative antibiotic drops. In Bulgaria, meticulous incision construction, hydration, and sealing are key components of intraoperative asepsis, and intracameral cefuroxime is often combined with a subconjunctival dexamethasone injection. In some cases, Dexachlor eye drops are also applied and used to cover the eye.

Across these settings, there is a shared understanding that careful surgical preparation, consistent use of povidone-iodine, and intracameral antibiotic administration can provide robust protection against intraoperative and early postoperative infections. However, specific measures vary depending on the surgeon’s clinical judgment, the costs and availability of certain drugs, and the need for more intensive prophylaxis when follow-up care may be limited. Although Georgia’s approach to intraoperative management reflects local constraints in postoperative follow-up, it is not based on increased antibiotic use. Instead, infection prevention relies primarily on meticulous antisepsis with povidone-iodine, combined with careful wound construction and controlled use of intracameral agents. Over a 15-year period and more than 20,000 cataract surgeries, only 2 cases of Gram-positive endophthalmitis were observed, representing an exceptionally low infection rate (~0.01%) [13]. This experience underscores that rigorous antiseptic practice can achieve excellent outcomes even when follow-up resources are limited. The principle of adapting perioperative protocols to local conditions can be extrapolated to other remote or underserved regions, where optimizing infection prevention and patient education during the initial surgical encounter is critical to ensuring postoperative safety.

### 2.3. Postoperative Management

Postoperative management of cataract surgery in the countries reviewed primarily focuses on prophylaxis with broad-spectrum topical antibiotics, typically fluoroquinolones, combined with anti-inflammatory agents. However, the duration and specific regimens vary slightly between countries. In most cases, third- or fourth-generation fluoroquinolones are employed, except for Romania, where the second-generation agent ciprofloxacin is preferred. The longest antibiotic regimen was reported in Czechia, where prophylaxis extends up to 3 weeks. In Georgia, a 10-day antibiotic course is followed, while in the remaining countries, antibiotics are usually administered for 7 days.

Other antibiotics occasionally employed postoperatively include aminoglycosides and chloramphenicol. Following antibiotic prophylaxis, corticosteroids—primarily dexamethasone—are administered for durations ranging from 7 days to 4 weeks. In some protocols, corticosteroid therapy continues for up to 21 days. Additionally, nonsteroidal anti-inflammatory drugs (NSAIDs) are prescribed for selected patients, typically for 2 to 6 weeks.

Rather than administering fluoroquinolones and corticosteroids separately, most experts recommend a fixed-dose combination (FDC) of these agents. The most common FDC is levofloxacin and dexamethasone, although other combinations—such as tobramycin with dexamethasone, netilmicin with dexamethasone, and chloramphenicol with betamethasone—are also employed. Levofloxacin in combination with dexamethasone is routinely administered 4 times a day for 7 days, while in Georgia, a more intensive regimen is used, with the FDC given 5 times a day for 14 days.

In Slovakia, fluoroquinolones (ofloxacin, levofloxacin, or moxifloxacin), aminoglycoside antibiotics (gentamicin, neomycin, or tobramycin), and other antibiotics are used postoperatively, often in combination with NSAIDs (e.g., nepafenac, diclofenac, or bromfenac) or with corticosteroids in high-risk patients, including those with a history of uveitis, diabetic retinopathy, or complicated surgery. While extended courses are sometimes recommended for complex cases, many specialists in Slovakia consider a short course sufficient, even for patients with diabetic retinopathy. Consequently, the combination of levofloxacin and dexamethasone is often administered for 7 days postoperatively.

Similarly, experts in Hungary recommend postoperative topical antibiotics, preferably third- and fourth-generation fluoroquinolones, for approximately 1 week, or the use of FDC eye drops containing both an antibiotic and a corticosteroid. These medications are recommended for sutureless clear corneal wound phacoemulsification. In some patients (such as those with diabetes mellitus) or to prevent postoperative inflammatory complications, nonsteroidal or corticosteroid eye drops are used for 2 to 6 weeks. In Bulgaria, many ophthalmologists prescribe fluoroquinolone drops (e.g., levofloxacin) in combination with dexamethasone, often as an FDC. Dexamethasone—preferably preservative-free, if available—can be continued after discontinuation of the antibiotic drops, with the total duration of the postoperative therapeutic regimen lasting around 20 days. These regimens are tailored to each patient’s individual risk factors, and patients are closely monitored through planned follow-up visits on postoperative days 1, 5–7, 21, and 45. Additional follow-up visits are scheduled for patients with underlying glaucoma or those considered at risk for poor compliance. Printed instructions and personal counseling are commonly provided to support adherence and ensure that patients understand the postoperative regimen and recognize when to seek immediate medical attention.

In Armenia, there is a growing trend towards using a combined antibiotic–corticosteroid agent administered for 1 week. This regimen is expected to replace the previous approach of prescribing the two medications separately.

In the Czech Republic, topical levofloxacin is routinely administered during the early postoperative period. In contrast, in Slovenia, many surgeons recommend FDC eye drops containing an antibiotic and a corticosteroid for 5 to 7 days, occasionally extending therapy to 14 days. This is typically followed by nonsteroidal therapy for approximately 3 weeks, unless contraindicated by corneal pathology. In certain high-risk cases, therapy with NSAIDs may be prolonged for up to 3 months.

Italy has adopted a regimen based on a national consensus, coordinated by the Italian Association of Cataract and Refractive Surgery, which supports the administration of a high-potency corticosteroid in combination with a broad-spectrum antibiotic [14]. Most experts agreed on a 7-day antibiotic course, after which patients are reassessed, and further therapy is tailored as needed.

In Poland, guidelines recommend short-term antibiotic prophylaxis, preferably with levofloxacin administered for 7 days [11,15]. However, topical steroids are generally continued for a longer duration—usually 2 to 4 weeks. In clinical practice, the use of an FDC of levofloxacin and dexamethasone is permitted, typically applied 4 times a day for 7 days, followed by topical corticosteroids alone.

In Romania, most specialists avoid initiating treatment with older agents such as chloramphenicol or tobramycin due to their higher associated risk of microbial resistance. Instead, levofloxacin is preferred for its continued efficacy. Surgeons generally initiate topical corticosteroid therapy on the day after surgery, with doses gradually tapered over a 20-day period. Third-generation fluoroquinolones are administered for 10 days, corticosteroids for 3 weeks with gradual reduction, and NSAIDs for 3 weeks. Hyperosmotic agents are added as needed. When an FDC of levofloxacin and dexamethasone is applied, the treatment duration is extended to 14 days.

Despite differences in treatment duration and specific drug choices, all approaches emphasize infection prevention through the use of an effective broad-spectrum antibiotic tailored to the patient’s individual risk factors. In this context, the term “tailored to the patient profile” refers to individualized decision making based on recognized clinical risk factors rather than formalized scoring systems. Across the countries analyzed, no standardized risk-stratification models for cataract surgery prophylaxis are currently employed. Instead, prophylactic decisions are guided by surgeon experience and patient-specific characteristics such as diabetes mellitus, ocular surface disease (e.g., blepharitis, dry eye), prior ocular surgery, uveitis, immunosuppression, or complicated cataract extraction. These factors influence the intensity, duration, and composition of antimicrobial and anti-inflammatory therapy. The development of validated ophthalmic risk-assessment tools could help harmonize prophylactic strategies and support evidence-based personalization of care across clinical settings. Many specialists favor short-term fluoroquinolone use, often combined with corticosteroids, but may extend or adjust therapy in patients with comorbidities such as diabetes, previous ocular surgery, or a complicated intraoperative course. The variation between shorter and more prolonged regimens largely reflects practical considerations, primarily the availability of follow-up care and the perceived need for enhanced prophylaxis.

## 3. Discussion

Although the risk of severe intraocular inflammation following modern cataract surgery is relatively low, the potentially serious consequences of endophthalmitis highlight the importance of minimizing its occurrence. The incidence of endophthalmitis can be reduced through a combination of strategies, including thorough preoperative patient assessment, appropriate surgical preparation, strict antiseptic protocol, precise surgical technique, and the judicious use of antimicrobial agents [16,17]. This last component is especially important given the growing global threat of AMR. While European guidelines on cataract surgery and endophthalmitis prevention are available [17,18], some countries have developed their own national recommendations, whereas others rely on clinical practices adapted to local settings in the absence of formal national guidelines. Differences between national practices and European Society of Cataract and Refractive Surgeons (ESCRS) recommendations across countries such as Poland, Romania, Slovakia, Hungary, Bulgaria, Czechia, Georgia, Armenia, and Slovenia likely reflect multifactorial influences. These include differences in local epidemiological patterns, healthcare infrastructure, regulatory requirements, and the availability or approval status of specific antimicrobial agents. In certain settings, additional constraints such as hospital procurement policies, reimbursement frameworks, or limited access to commercially prepared intracameral antibiotics may constrain full implementation of ESCRS recommendations. Furthermore, variations in aseptic standards, surgical volume, and institutional protocols, as well as differences in clinician training and patient adherence, can contribute to these discrepancies. Such diversity underscores that while European guidelines provide a robust framework, their successful application requires parallel efforts to improve education, ensure equitable drug access, and enhance aseptic conditions within surgical facilities.

The heterogeneity observed among countries also reflects the reality that strict adherence to general guidelines may not always be optimal. Geographic variations in AMR patterns, differences in clinical resources and patient population characteristics necessitate a degree of flexibility. Moreover, official recommendations primarily cover the fundamental aspects of surgery, leaving finer procedural details to the discretion of individual surgeons. Their choices are shaped by clinical experience, available resources, personal preferences, and patient-specific needs and expectations. The growing body of published evidence further influences these decisions, often prompting continuous adaptation of prophylactic protocols.

Ophthalmic-specific AMR surveillance remains limited in Eastern Europe, but available national data highlight significant resistance challenges that affect both antibiotic prophylaxis and treatment. In Armenia, for example, the Institute for Health Metrics and Evaluation reported a measurable AMR burden, with hundreds of deaths annually attributed to resistant infections [19,20]. National efforts to address this include the AMR National Action Plan and initiatives supported by the WHO aimed at increasing awareness and improving screening despite ongoing challenges such as rising antibiotic consumption, over-the-counter availability, and limited laboratory capacity [19,20]. Similarly, in Georgia, the National Strategy for Combating AMR (2017–2020) and WHO TrACSS 2021 profile reveal high usage of broad-spectrum antibiotics, along with significant gaps in surveillance and antimicrobial stewardship. These programs, coordinated by the National Center for Disease Control and Public Health with support from the WHO and CDC, underscore the importance of aligning ophthalmic antibiotic practices with local resistance trends and strengthening regional AMR surveillance to enable more evidence-based prophylactic strategies [21].

From a procedural standpoint, preoperative prophylaxis focuses on reducing ocular surface microbial load and ensuring optimal antisepsis before incision. However, this study revealed the persistence of substantial variability regarding the use of preoperative topical antibiotics. This variability largely reflects a growing body of evidence suggesting limited or no additional benefit from preoperative topical antibiotic prophylaxis [22,23,24]. Furthermore, recent cost-effectiveness analyses indicate that preoperative topical antibiotic use may not be economically justified compared to no antibiotic use for the prevention of postoperative endophthalmitis [25]. The ESCRS guidelines acknowledge the lack of strong evidence supporting routine preoperative topical antibiotic use, leaving such decisions to clinical judgment. Consequently, no universally accepted approach to preoperative prophylaxis has been established.

Concerns regarding the administration of topical antibiotics 2 to 3 days before surgery include potential disruption of the ocular surface microbiome and the risk of promoting AMR. For this reason, some countries rely predominantly on topical antimicrobials or antiseptics, emphasizing the efficacy of rigorous surgical preparation and minimizing unnecessary antibiotic exposure. Conversely, some clinicians advocate for preoperative antibiotic drops to reduce bacterial load and prepare the ocular surface for surgery. In selected settings, NSAIDs may be started pre-operatively for specific indications, though this is not routine.

Intraoperatively, antibiotic administration within the anterior chamber remains the cornerstone of endophthalmitis prevention. Common element of endophthalmitis prophylaxis in several of the countries studied is the use of intracameral cefuroxime. This broad-spectrum cephalosporin antibiotic acts by disrupting bacterial cell wall synthesis, resulting in cell lysis and death. Its widespread use is supported by the pharmacokinetic and pharmacodynamic properties, as cefuroxime remains effective in inhibiting pathogen growth for up to 4 to 5 h after surgery, thereby providing coverage during the critical intraoperative period [26]. Cefuroxime, a second-generation cephalosporin, exhibits bactericidal activity that is both concentration- and time-dependent. It is effective against the most common Gram-positive pathogens implicated in postoperative endophthalmitis, such as *Staphylococcus epidermidis* and *Streptococcus pneumoniae*, although it lacks activity against methicillin-resistant strains—*S. aureus* (MRSA) and *S. epidermidis* (MRSE)—as well as *Enterococcus faecalis* and *Pseudomonas aeruginosa* [27]. Its use gained widespread acceptance following the landmark ESCRS study [28], which demonstrated a 5-fold reduction in endophthalmitis rates with a single 1 mg intracameral injection administered at the end of cataract surgery. These findings have been confirmed by subsequent studies [29,30,31] and are consistent with recommendations from several professional bodies, including the National Institute for Health and Care Excellence (NICE) 2017 and ESCRS [17].

Concerns have been raised that a single perioperative intracameral dose of cefuroxime may not provide sufficient antibacterial coverage throughout the entire wound healing period, given its bactericidal activity is both concentration- and time-dependent. Nevertheless, some studies showed that intracameral antibiotics alone may be as effective as combined intracameral and topical antibiotics regimens [23,32]. Cefuroxime remains one of the most commonly used intracameral agents. Alternatives such as intracameral moxifloxacin or vancomycin offer broader antimicrobial spectra; however, concerns about ocular toxicity limit their routine use [23]. Intracameral moxifloxacin is not universally approved or available as a ready-to-use formulation, and in many regions only off-label or compounded preparations can be used, raising issues related to sterility and dosing consistency. Consequently, European guidelines recommend intracameral cefuroxime as first-line prophylaxis, reserving moxifloxacin mainly for patients with beta-lactam allergy [17,18].

Use of intracameral vancomycin has declined following reports of hemorrhagic occlusive retinal vasculitis, a rare but severe vision-threatening complication [33,34]. Furthermore, registry data from countries such as Sweden indicate no increase in endophthalmitis rates when postoperative topical antibiotics are omitted in eyes receiving intracameral cefuroxime [35].

One consistent perioperative element in all settings is the use of povidone-iodine as a standard antiseptic in cataract surgery. This widely adopted practice is supported by robust evidence demonstrating the efficacy of iodine-based preparations in reducing bacterial contamination and lowering the incidence of postoperative endophthalmitis [36]. It also aligns with international guidelines, including those issued by the ESCRS [17] and NICE [37]. Povidone-iodine acts by releasing free iodine, which readily penetrates bacterial membrane and leads to cell death. Effective concentrations range from 0.005% to 10%. Interestingly, the release of free iodine becomes less efficient as the concentration of povidone-iodide increases; dilution of the solution facilitates iodine release. Lower concentrations (0.1–1.0%) require shorter contact times, while higher concentrations (2.5–10%) necessitate longer exposure to achieve microbial kill and are therefore typically used for single-application purposes, such as eyelid skin disinfection or intravitreal injections. Although no true genetic resistance to povidone-iodine has been documented [38,39], its antimicrobial efficacy can vary depending on microbial and environmental factors. For example, *Acinetobacter* species demonstrate reduced susceptibility due to the high mycolic acid content in their cell walls, which limits iodine penetration [40]. Additionally, the activity of povidone-iodine varies depending on the bacterial state: it is highly effective against biofilm-associated bacteria at low concentrations but less active against planktonic cells at the same levels [41]. Its performance is also influenced by dilution and contact time. From a clinical standpoint, some patients exhibit ocular intolerance, including surface irritation or allergic reactions, necessitating consideration of alternatives such as 0.05% aqueous chlorhexidine. Although the draft version of the ESCRS guidelines for cataract surgery [18] permits repeated rinsing of the ocular surface every 20 to 30 s with 0.25% povidone-iodine drops, the preferred approach in the countries analyzed involves the application of 5–10% povidone-iodine drops for 3 min prior to the procedure. In cases where povidone-iodine is contraindicated, 0.05% aqueous chlorhexidine may be used as an alternative. In some countries, such as Bulgaria, a 5% povidone-iodine solution is used exclusively, primarily due to concerns about potential toxicity at higher concentrations. In contrast to the universal consensus on antisepsis, the role of topical antibiotic prophylaxis before surgery remains controversial.

Postoperative management focuses on maintaining antibacterial protection during healing and controlling inflammation to optimize recovery. Postoperative antibiotic use seems to be more widely accepted, likely as a means to complement the time-dependent activity of intracameral cefuroxime, which may not provide prolonged antibacterial coverage throughout the entire wound healing period. In postoperative contexts, fluoroquinolone eye drops remain the preferred agents due to their broad antimicrobial spectrum, partial corneal epithelium penetration, and widespread commercial availability. Fluoroquinolones are the first fully synthetic class of antibiotics, exerting their antibacterial effects by inhibiting topoisomerase II, topoisomerase IV, and DNA gyrase—enzymes essential for bacterial chromosome replication and function [42]. Third- and fourth-generation fluoroquinolones, which are among the most widely used agents in cataract surgery prophylaxis, retain the broad-spectrum activity against Gram-negative bacteria seen in earlier generations, while exhibiting enhanced potency against Gram-positive organisms. This is particularly important, as Gram-positive bacteria continue to be the most frequently identified pathogens in postoperative endophthalmitis [43,44]. These newer-generation agents also demonstrate improved penetration into the anterior segment of the eye and a lower tendency to induce AMR compared to older antibiotics [45,46]. Such characteristics are especially valuable in the context of increasing resistance to multiple antibiotic classes, including earlier fluoroquinolones, cephalosporins, and aminoglycosides [44,47,48]. However, although newer-generation fluoroquinolones were initially considered less likely to promote resistance due to their dual inhibition of DNA gyrase and topoisomerase IV, recent evidence indicates that resistance is still emerging rapidly, particularly with widespread or prophylactic use [49,50]. Regional differences influence the selection of topical fluoroquinolones. In many countries, levofloxacin remains one of the most frequently applied agents, primarily due to its broad antimicrobial spectrum, improved efficacy against Gram-positive bacteria and atypical pathogens, favorable ocular tissue penetration, and well-established safety profile [51,52]. Its popularity also reflects high commercial availability and lower resistance rates compared to older fluoroquinolones. Some countries (Czechia, Georgia, Hungary) prefer higher-generation fluoroquinolones such as moxifloxacin or gatifloxacin since they, offer broader antimicrobial coverage and, in some settings, lower resistance rates. These variations highlight the importance of local AMR patterns, drug accessibility, and cost considerations in guiding antibiotic selection for cataract surgery prophylaxis.

Postoperative anti-inflammatory therapy, alongside antimicrobial prophylaxis, also plays a critical role in optimizing visual recovery and minimizing complications after cataract surgery. Topical NSAIDs are widely prescribed for 2–4 weeks following surgery to suppress inflammation and reduce the risk of cystoid macular edema. The use of NSAIDs for approximately 3 weeks is common across many European countries and is supported by clinical evidence demonstrating reduced edema rates, particularly in patients with diabetes, uveitis, or complicated surgeries [53,54,55]. These agents may also alleviate postoperative discomfort and accelerate visual rehabilitation. However, practice patterns vary, with some countries prescribing NSAIDs routinely for all patients, whereas others limiting their use to high-risk cases or combinations with corticosteroids. The duration and choice of agent (e.g., nepafenac, bromfenac, ketorolac) often depend on local protocols, surgeon preference, drug availability, and reimbursement policies. Although a 3-week regimen has become conventional, it is often rooted in tradition rather than uniform evidence, as some recent studies suggest that shorter courses may offer comparable efficacy in uncomplicated cases. These variations highlight the need for further comparative research to define the optimal nonsteroidal anti-inflammatory regimen and to harmonize practice based on patient risk and clinical outcomes [53,54,55].

While non-antibiotic technologies are not yet part of routine practice, several promising approaches are under investigation. Silver nanoparticles and other nanomaterials show antimicrobial activity in ocular applications; however, concerns regarding toxicity and limited translational data remain significant challenges to their clinical use [56]. Ozone therapy has shown some experimental antimicrobial efficacy, but clinical evidence supporting its use in ophthalmic surgery is currently minimal [57]. Meanwhile, sustained-release implants, hydrogels, and nanoparticle-based delivery systems (such as solid lipid nanoparticles and microneedles) are being developed to provide controlled release of antibiotics or anti-inflammatory agents, potentially reducing the need for frequent perioperative dosing [58]. These innovations warrant continued evaluation as potential adjuncts to standard antiseptic and antibiotic protocols.

The findings of this study, which reveal marked differences in prophylactic antibiotic practices across countries, highlight the need for strengthened antibiotic stewardship in ophthalmic care. These observations align closely with the WHO Global Action Plan on AMR [59], which calls for optimizing antimicrobial use, enhancing surveillance, and reducing infection incidence through improved preventive measures. Integrating ophthalmic antibiotic prophylaxis into national and regional AMR stewardship programs, ensuring the systematic collection of resistance data specific to ocular pathogens, and promoting international collaboration to harmonize prophylactic practices are essential steps toward achieving these goals. Strengthening the link between ophthalmic practice and global AMR frameworks will help sustain the long-term safety and efficacy of cataract surgery worldwide [60]. Within this broader stewardship context, pharmacologic optimization also plays a key role. Modern fluoroquinolones, such as levofloxacin, are designed to provide broad-spectrum antimicrobial coverage while maintaining a favorable safety profile and a lower risk of resistance development [52]. The emergence of FDCs (e.g., levofloxacin combined with dexamethasone) offers an opportunity to shorten of a postoperative treatment course without compromising efficacy. Recent studies have shown that this topical combination allows for a shorter treatment duration, demonstrating that a 1-week course can be as effective as the traditional 2-week regimen. This reduced schedule not only minimizes the incidence of corticosteroid-related adverse effects but also helps limit the development of AMR while maintaining effective postoperative infection control [61,62]. However, commercial factors, including drug pricing and cost-effectiveness, may influence the adoption and accessibility of FDCs in clinical practice. Although available evidence generally suggests the cost-effectiveness of FDCs [63], comprehensive economic evaluations specifically focused on their use in cataract surgery are lacking. Comprehensive health-economic evaluations would provide valuable insights into the broader feasibility and sustainability of incorporating FDCs into routine ophthalmic care.

At the clinical level, international guidelines, including those from the ESCRS, NICE, and the American Academy of Ophthalmology, generally converge on several key principles: thorough antisepsis, judicious antibiotic use, and the incorporation of anti-inflammatory therapy for selected patients [17,37,64,65]. These principles highlight a broader challenge: the need for continuous monitoring of emerging resistance patterns and systematic collection of local epidemiological data. In this context, adopting a flexible, patient-specific approach to prophylaxis is essential. Based on current evidence, effective strategies to reduce AMR should prioritize strict adherence to sterile surgical protocols, routine use of povidone-iodine for antisepsis, avoidance of prolonged antimicrobial courses, and reduced use of prophylactic antibiotics in uncomplicated procedures [16].

Emerging microbial resistance remains a critical global health challenge, and the increasing resistance to fluoroquinolones is particularly concerning. These agents are among the most widely prescribed first-line antibiotics in human medicine and also play a key role in veterinary practice, making their declining efficacy a major threat to both public health and surgical safety, as it jeopardizes both infection prevention in surgical settings and the treatment of common bacterial infections [59,66]. In ophthalmology, fluoroquinolones are widely used for postoperative prophylaxis following cataract surgery. While their broad spectrum and effectiveness make them attractive options, frequent and prolonged use, especially without consideration of local resistance patterns, can accelerate the emergence of resistant ocular pathogens. Increasing ciprofloxacin resistance among pathogens such as *E. coli*, *S. aureus*, and *P. aeruginosa* limits is utilization [67], underscoring the importance of continuous AMR surveillance and consideration of newer-generation fluoroquinolones as access improves. Local surveillance data indicate acceptable susceptibility of ocular pathogens to ciprofloxacin, supporting its continued use [68,69].

Beyond microbiological considerations, the environmental impact of antibiotic overuse warrants increasing attention. Topical antibiotics, when excreted through tears or disposed of improperly, can enter wastewater and surface water systems, contributing to the persistence and dissemination of AMR genes among environmental bacteria [70,71,72]. This environmental reservoir of resistance can ultimately affect both human and animal health, underscoring the importance of responsible prescribing practices and proper disposal of unused medications. As awareness of these ecological consequences grows, future ophthalmic and public health guidelines may begin to incorporate environmental stewardship principles as part of comprehensive strategies to combat AMR.

Finally, patient adherence remains a pivotal determinant of prophylactic success. Simplified combination eye drops, such as antibiotic-steroid formulations, may improve compliance while potentially limiting overall antimicrobial exposure. Such approach aligns with global recommendations for rational antibiotic use and represents a practical pathway toward sustainable ophthalmic antimicrobial stewardship [64].

### Limitations

This study has several limitations. It was restricted to describing current clinical practices and approaches used for infection prevention in cataract surgery. Data on patient outcomes, satisfaction, compliance, and adverse events were not collected or analyzed, as the contributing experts reported practice patterns rather than individual-level clinical outcomes. Although these factors are highly relevant for assessing the real-world effectiveness of prophylactic strategies, their inclusion would have considerably increased the length and complexity of the manuscript and was therefore beyond the scope of this work. Future studies incorporating clinical outcomes and patient perspectives would provide valuable context to complement the descriptive findings presented here.

Although differences in clinical practice were observed between countries, these variations are unlikely to be primarily driven by economic factors, as all participating nations are classified by the World Bank as upper-middle- or high-income economies. Consequently, the findings may not be fully generalizable to low- and middle-income countries, where limited access to pharmaceuticals, diagnostic capacity, and surgical infrastructure can significantly influence infection-prevention practices and clinical decision making. Broader inclusion of data from low- and middle-income countries in future studies would help capture these important contextual differences.

The selection of countries in this study was based on voluntary participation by contributing experts rather than a predefined geographic framework. As a result, the sample is predominantly Euro-centric, which may limit generalizability. Nevertheless, the data provide a valuable snapshot of regional practices and highlight the importance of expanding future collaborations to include additional countries and non-European settings.

## 4. Materials and Methods

**Figure 2:**
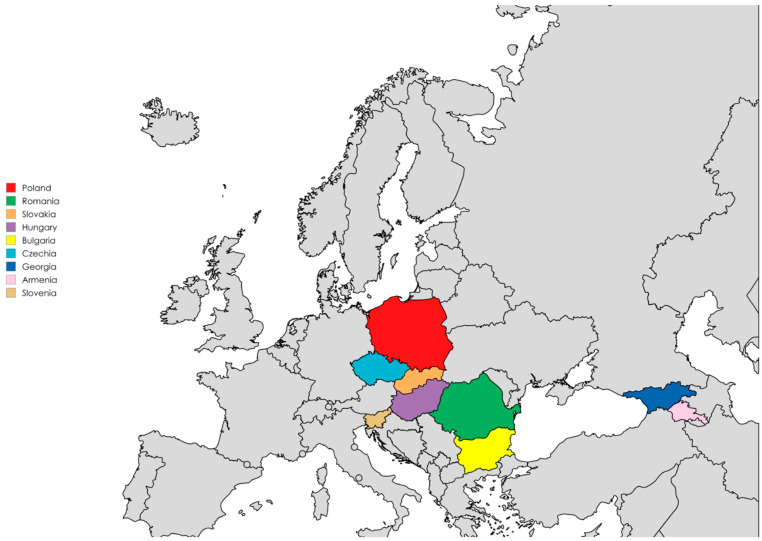
Countries represented by the contributed experts, highlighted in color (map created with https://www.mapchart.net/, accessed on 20 July 2025).

Ophthalmology experts from 10 countries (Figure 2) contributed insights to national approaches for infection prevention in the context of cataract surgery. Where official recommendations were available, these formed the basis of the input; in their absence, expert opinion and clinical practice informed the responses. Each expert was invited to provide a detailed written description of standard national practices for infection prevention in the context of cataract surgery, structured across the three stages: preoperative, intraoperative, and postoperative management. Data collection did not involve structured questionnaires or formal interviews; instead, information was obtained through email correspondence and document exchange. The aim was to document representative national practices as reported by experienced clinicians, rather than to conduct a reproducible, survey-based study.

Although this study was categorized as a review, its methodology differs from that of a systematic or scoping review. The primary aim was to synthesize available national and regional data on endophthalmitis prophylaxis while integrating insights from experienced clinicians representing diverse healthcare systems. As such, the work should be regarded as a narrative review supported by expert opinion, rather than a reproducible, protocol-driven review. This hybrid approach was chosen to provide both an overview of existing recommendations and a realistic perspective on their implementation in daily practice, capturing the variability between formal guidelines and real-world application across countries.

## 5. Conclusions

This study highlights both common practices and notable national differences in antibiotic prophylaxis strategies for preventing endophthalmitis following cataract surgery. Despite some variations, several consistent themes emerge: the essential role of povidone-iodine in preoperative antisepsis, the widespread adoption of intracameral cefuroxime to reduce postoperative infection risk, and the continued emphasis on prudent antibiotic use to minimize the development of AMR. The use of preoperative topical antibiotics varies across countries—some incorporate them routinely, while others omit them entirely. In contrast, postoperative prophylaxis tends to be more consistent, with most countries favoring fluoroquinolone eye drops, although treatment duration varies considerably. These differences reflect country-specific national guidelines, resistance profiles, and healthcare system capacities.

The observed diversity of approaches also underscores the need for evidence-based standardization and harmonization of prophylactic protocols across Europe and beyond. At the same time, effective strategies must remain responsive to local microbiological environments, resistance patterns, and patient populations, ensuring flexibility within standardized frameworks.

Future research should prioritize multicenter, prospective studies designed to rigorously evaluate the safety, efficacy, and cost-effectiveness of various prophylactic regimens, while also incorporating patient-centered outcomes, including adherence, satisfaction, and adverse events. Additionally, strengthening AMR surveillance specific to ocular pathogens and integrating ophthalmic antibiotic stewardship into national and regional AMR programs will be critical. Finally, international collaboration among professional societies, regulatory bodies, and public health organizations will be key to translating evidence into coherent practice standards.

Sustained vigilance, personalized care, and continued research on resistant pathogens are essential to preserve the safety and efficacy of cataract surgery in an era of evolving microbial resistance and expanding global surgical demand.

## Data Availability

Data is contained within the article or Supplementary Materials.

## Supplementary Materials

The following supporting information can be downloaded at: https://www.mdpi.com/article/10.3390/antibiotics14121192/s1, Supplementary Table S1. Expert-reported national prophylactic antibiotic, anti-inflammatory and other practices in cataract surgery across selected European countries, plus Armenia and Georgia.

## Abbreviations

The following abbreviations are used in this manuscript:

AMR	antimicrobial resistance
ESCRS	European Society of Cataract and Refractive Surgeons
FDC	Fixed-dose combination
MRSA	methicillin-resistant *S. aureus*
MRSE	methicillin-resistant *S. epidermidis*
NICE	National Institute for Health and Care Excellence
NSAIDs	non-steroidal anti-inflammatory drugs
