# Supplementary material for: Country-Specific Approaches to Preventing Infections in Cataract Surgery
Source: Antibiotics (Basel). 2025 Nov 23;14(12):1192. doi: 10.3390/antibiotics14121192 (PMC12729967; doi:10.3390/antibiotics14121192)
Supplement: Supplementary file 1 [file antibiotics-14-01192-s001.zip › antibiotics-3874242-supplementary.pdf]

Supplementary Table S1. Country-specific approach at various stages of infection prophylaxis in cataract surgery.

Country, reference	Management		
	Preoperative	Intraoperative	Postoperative
Armenia (expert's input)	- hyaluronic acid	- povidone-iodine<br>- subconjunctival dexamethasone 0.5 mL<br>- levofloxacin: 1 drop	- levofloxacin and dexamethasone as an FDC (1 drop, 4x/day, 7 days)
Bulgaria (expert's input)	- no antibiotic	- povidone-iodine<br>- intracameral cefuroxime<br>- subconjunctival dexamethasone	- levofloxacin and dexamethasone (preferably FDC)<br>- continuation with dexamethasone up to 3 weeks<br>- NSAIDs in selected patients
Czechia [1,2] and expert's input	- second- to fourth-generation fluoroquinolones, aminoglycosides, or other antibiotic combinations 1-3 days before surgery	- povidone-iodine: 5-10% for the conjunctival sac, 10% for periocular skin,<br>- intracameral cefuroxime: 1 mg in 0.1-ml solution (92%)<br>- different type of antibiotic intracamerally (6%)<br>- antibiotic drops only (2%)	- fluoroquinolones (ofloxacin, levofloxacin, moxifloxacin), aminoglycosides (neomycin, gentamycin, tobramycin), or other antibiotic combinations, possibly combined with NSAIDs and corticosteroids in specific cases, for 3 weeks following uncomplicated procedures
Georgia (expert's input)	- no antibiotics.<br>- NSAIDs 10 days prior to surgery only in cases of pseudoexfoliation syndrome	- Povidone-iodine: 10% for periocular skin (before draping), 5% for conjunctival sac (rinsed with BSS)<br>- 5% povidone-iodine at the end of surgery to check wound integrity, then flushed with BSS.<br>- 1 drop of third-generation fluoroquinolone (e.g., levofloxacin) instilled before applying the eye pad.	- third-generation fluroquinolones (4x/day, 10 days) and steroid drops (6x/day, tapered over 3 weeks);<br>- NSAIDs (3x/day, 20 days)
Hungary [3] and expert's input	- no antibiotic	- povidone-iodine: 5% for the conjunctival sac, 10% for periocular skin,<br>- intracameral cefuroxime (0.1 ml of 1% solution)	- third- and fourth-generation fluoroquinolones (7 days) or fluoroquinolones with corticosteroids as an FDC (7 days)<br>- NSAIDs in selected patients for 2-6 weeks

Italy [4] and expert's input	- no antibiotic	- povidone-iodine: 5% on the ocular surface, 5–10% in the periocular skin<br>- intracameral cefuroxime	- topical antibiotics (aminoglycosides, chloramphenicol, and quinolones – preferably levofloxacin) for 7 days<br>- corticosteroid (dexamethasone and betamethasone) for 7 days<br>- antibiotic/corticosteroid as an FDC (tobramycin/dexamethasone, netilmicin/dexamethasone, chloramphenicol/betamethasone, and levofloxacin/dexamethasone) – 1 drop 4x/day for 7 days
Poland [5] and expert's input	- optional use of topical antibiotic drops (fluoroquinolones) 2 days before surgery	- povidone-iodine: 5% for the conjunctival sac, 10% for periocular skin,<br>- intracameral cefuroxime: 1mg in 0.1ml solution	- broad-spectrum fluoroquinolone for 7 days (preferably levofloxacin)<br>- NSAIDs for 4-6 weeks<br>- corticosteroids for 2-4 weeks (preferably loteprednol or dexamethasone)
Romania (expert's input)	- no antibiotics	- no antibiotic	- immediately after surgery Tobradex eye drops applied once<br>- levofloxacin with dexamethasone as an FDC for 7 days
Slovakia (expert's input)	- a combination of levofloxacin and dexamethasone 1 day before surgery	- povidone-iodine: 3-5% for the conjunctival sac, 5-10% for periocular skin,<br>- intracameral cefuroxime 1 mg in 0.1-ml solution	- fluoroquinolone, aminoglycoside, other antibiotic combinations, or levofloxacin with dexamethasone as an FDC (7 days)<br>- NSAIDs<br>- corticosteroids in selected patients
Slovenia (expert's input)	- a combination of an antibiotic with dexamethasone and a NSAIDs 3 days before surgery	- povidone-iodine: 5%	- levofloxacin and dexamethasone as an FDC (5-7 days)<br>NSAIDs (3 weeks)

FDC, fixed dose combination; NSAIDs, non-steroidal anti-inflammatory drugs
